# Dementia Care under COVID-19 and Infectious Disease Pandemic Restrictions

**DOI:** 10.1093/geroni/igaa057.3448

**Published:** 2020-12-16

**Authors:** Kelly Bradbury, Elaine Moody, Katie Aubrecht, Meaghan Sim, Melissa Rothfus

**Affiliations:** 1 Dalhousie University, Halifax, Nova Scotia, Canada; 2 St. Francis Xavier University, Antigonish, Nova Scotia, Canada

## Abstract

Emergency measures including social distancing and program restrictions during COVID-19 has reduced supports for people living with dementia and family/friend caregivers in the community. Consequently, these reductions in dementia services and resources have added to existing challenges and (in)equities for this stigmatized population. The objectives of this study were to identify how community-based resources and services for people with dementia and their caregivers are impacted by public health emergency measures enacted during COVID-19 and other infectious pandemics and secondly, use an intersectional health equity perspective to explore how supports for people and families living with dementia are affected by social determinants of health. A scoping review using JBI methodology was conducted. Academic databases searched included Embase, Medline, CINAHL and PAIS. Grey literature was searched using the CADTH tool. English articles published after 2000 in high-income countries were included. Data was extracted by two reviewers using an adaptation of the Health Equity Impact Assessment tool to explore factors related to health equity. Findings included articles discussing the COVID-19 pandemic (N=15). Most alterations to dementia services included switching to telehealth platforms with some advantages/disadvantages of this method discussed. Limited information on how different populations experienced service changes was identified and more research is needed to address issues of (in)equities for people living with dementia and their caregivers during public health emergencies. Information on how health emergency responses affects dementia services and their users will provide important information on resources for current and future efforts to analyze and assess their impacts.

